# Hospital Meetings

**Published:** 1906-02-17

**Authors:** 


### HOSPITAL MEETINGS.

### ST. MARK'S HOSPITAL.

The annual general meeting of the donors and subscribers of this hospital was held at the Mansion House on February 8, the Lord Mayor being in the chair. The report of the Committee of Management having been adopted, the meeting proceeded to pass the usual votes of thanks. Mr. T. Mark Merriman, member of the House Committee, in proposing a vote of thanks to the medical officers for the skilful and gratuitous discharge of their duties during the past year, dwelt upon the harmony that existed between the medical staff and the Committee of Management, but remarked that there was one vexed question between them on the subject of the payments of patients, some of the staff being rather sensitive lest it should be said that these payments were made for their services.

Mr. F. S. Edwards, senior hon. surgeon, in responding, said that it was a cause of great gratification to the staff to know that nearly 100 more operations had been performed during the past year than ever before. There had been nearly 100 more in-patients, which practically meant the same increase in operations. This was indeed a record, especially when it was borne in mind that the total was only 600. There had also been an increase in the number of out-patients, the only decrease being in the number of out-patients' attendances. This was probably due to the question of the payments of patients. If a patient had to pay when there was very little the matter with him, he would be likely to think twice about coming to the hospital.

Sir R. Biddulph Martin, treasurer, in replying to a vote of thanks for his services, said that those who had founded St. Mark's Hospital had been actuated by the wish that special treatment should be available to the poor as well as to the rich. With regard to the payment of patients, a great difficulty had arisen. It had been felt for some years that many of those attending the out-patients' department were not entitled to receive assistance, and were doing so to the detriment of the really poor. It was not right that any hospital should cut out the medical practitioners in their neighbourhood; they should rather be made almoners to the hospital, and help them to discover which were the really deserving cases. He hoped that this question would not be lost sight of in the future. At St. Mark's they were most anxious to give the utmost amount of real charity to those who were deserving of it.

A vote of thanks to the Lord Mayor for presiding terminated the proceedings.

### LONDON FEVER HOSPITAL.

The annual general meeting of governors was held at the Hotel Victoria on February 9, the chair being taken by Judge Lumley Smith.

Mr. Norman, who moved the adoption of the statement of receipts and expenditure for 1905, said that the past year appeared to have been satisfactory financially. Their total ordinary expenditure was £600 less than in 1904, and their total ordinary income £338 more. They had been obliged to spend £1,410 on building improvements, and to meet this charge had been compelled to use a good deal of their invested funds. The committee trusted to the generosity of the public to enable them to replace the investments.

Judge Lumley Smith, having delivered a message from Lord Balfour of Burleigh, their President, who much regretted his inability to take the chair on account of a slight injury, went on to say that during the past year the number of patients had been rather less than it had been for the past twenty years—only 475 against 606 in the previous year. Towards the end of the year an outbreak of scarlet fever, combined with the rebuilding, had caused great pressure on their space, but they had not been compelled to refuse many cases. The death-rate during 1905 had been very low. The object of the hospital was to provide for a class of patients who were able and willing to pay a little for themselves and did not wish to go to those hospitals which were entirely supported out of the rates. During the last twenty years 12,000 patients had availed themselves of the hospital. After giving an analysis of the professions and classes to which the patients belonged, he went on to say that those people, if they had not been treated at the London Fever Hospital, would in all probability have been nursed in their own homes with a much greater risk of spreading infection. The hospital had been established 103 years, during which time 82,000 patients had been treated.

Lord Balfour of Burleigh having been again elected President, a vote of thanks to the chairman closed the meeting.

### GENERAL LYING-IN HOSPITAL, LAMBETH.

In our notice of the annual meeting of the General Lying-in Hospital last week the number of women attended in their own homes was incorrectly given as 259, instead of 2,059.

